# Legume substitution alone is not enough: A multi-criteria LCA of low-soybean meal broiler feed systems

**DOI:** 10.1016/j.psj.2026.107979

**Published:** 2026-10-17

**Authors:** Ashraf Alkhtib, Dawn Scholey, Mike Bedford, James Bentley, Emily Burton

**Affiliations:** a Nottingham Trent University, School of Animal, Rural, and Environmental sciences, Southwell, Nottinghamshire, NG25 0QF, UK; b AB Vista Feed Ingredients Ltd, Marlborough, UK; c Hubbard SAS, Mauguérand, Le Foeil, Quintin, 22800, France

**Keywords:** Soybean meal, Legume, Enzyme, Performance, Carbon footprint

## Abstract

The study evaluated the effect replacing soybean meal with UK-grown legumes and sunflower meal and enzyme supplementation on environmental impact of broilers. A total of 960 male broilers (Ross 308 and JA787) were assigned to five diets, high nutrient density (HN), moderate nutrient density (MN), a legume-based MN diet without enzymes (LEG), and LEG supplemented with standard (LEG-STD) or super-dose (LEG-S) NSP enzymes. In LEG diets, a mix of pea, bean, lupin, and sunflower meal at equal proportions replaced 50% of SBM in MN. The enzyme mix contained 0.01% phytase and 0.01% Signis, while the super-dose mix contained 0.05% phytase and 0.015% Signis. Pen-level life cycle assessment of consumed feed was normalised to meat yield (1 bird/pen at D35 for Ross 308 and D42 for JA787) with legumes produced under conventional or low fertilisation and plant protection input legume production scenarios. Impact categories included global warming potential with land use change (GWP), freshwater eutrophication (EUT), terrestrial acidification (ACID), ecotoxicity (ETOX), land use (LU), and water resource depletion (WRD). Data was analysed following 5 × 2 full factorial design. Legume based diets had significantly lower GWP compared to HN regardless of bird strain (P < 0.05) while meat yield was not significantly affected, however, these diets had significantly higher LU and ETOX compared to HN. When legumes were produced under low-input conditions, the difference between LEG-S and HN became insignificant (P > 0.05), however, the diet still have significantly higher ETOX (P < 0.05). These findings highlight trade-offs in feed-based mitigation and support integrated agronomic and nutritional strategies.

## Highlights

• Legume diets reduced broiler feed GWP without affecting meat yield.
• Legume diets increased land use and ecotoxicity impacts.
• Enzyme supplementation did not fully offset freshwater impacts.
• Low-input legume production reduced differences in GWP outcomes.
• Findings highlight trade-offs in feed-based environmental mitigation.

## Introduction

Poultry meat is projected to remain the most consumed animal protein globally, accounting for nearly 39% of total meat intake by 2030, driven by its affordability, nutritional value, and absence of cultural barriers (OECD, 2025). Feed formulation is central to the sustainability of poultry production, as feed ingredients contribute up to 70–75% of total production costs and represent major environmental hotspots in life cycle assessments (Attia et al., 2024). Soybean meal (SBM) has long been the dominant protein source in broiler diets due to its high crude protein content and favourable amino acid profile (Dalgaard et al., 2007) . However, reliance on SBM raises critical sustainability concerns, including deforestation risks linked to South American supply chains and significant greenhouse gas emissions associated with cultivation and transport.

Grain legumes such as faba bean (Vicia faba), field pea (Pisum sativum), and lupin (Lupinus spp.) are increasingly considered viable alternatives to SBM. These crops offer agronomic benefits including biological nitrogen fixation, improved soil health, and reduced synthetic fertiliser requirements (Jensen et al., 2011). Nutritionally, legumes provide substantial crude protein and metabolizable energy, though their amino acid profiles are less optimal than SBM, necessitating careful formulation and supplementation (Hejdysz et al., 2017).

A major constraint to high legume inclusion in poultry diets is their elevated non-starch polysaccharide (NSP) content, which increases digesta viscosity and impairs nutrient digestibility. Exogenous NSP-degrading enzymes, particularly xylanase, have been shown to mitigate these effects, improving energy utilisation, nitrogen retention, and feed conversion ratio (Kocher et al., 2000). Enzyme supplementation has also been shown to maintain broiler performance under nutrient-reduced diets while reducing nitrogen and phosphorus excretion (Chen et al., 2023). However, the environmental implications of substituting SBM with legumes remain complex. While legume-based diets can reduce feed-related greenhouse gas emissions, they may increase other impact categories such as land occupation, water consumption, acidification, and fresh water toxicity. Consequently, evaluating soybean meal substitution solely in terms of greenhouse gas emissions may overlook important environmental trade-offs. The magnitude of these trade-offs is influenced not only by feed formulation but also by the agronomic practises used to produce the alternative protein crops. Grain legumes can obtain part of their nitrogen requirement through biological nitrogen fixation, potentially reducing their dependence on synthetic nitrogen fertiliser. Opportunities may also exist to optimise phosphorus, potassium and plant-protection inputs according to soil nutrient status, crop requirements and local production conditions. Crop responses to phosphorus application depend strongly on initial soil phosphorus status (Ros et al., 2020), while improved potassium-management recommendations may help avoid unnecessary nutrient application (Brouder et al., 2021). Reduced-input production could therefore lower some environmental burdens associated with legume cultivation. Nevertheless, it remains uncertain whether such agronomic improvements are sufficient to offset the wider environmental trade-offs arising when legumes replace soybean meal in broiler diets. Therefore, this study aimed to evaluate the combined effects of UK-grown legumes and NSP enzyme supplementation on broiler growth performance and multi-dimensional environmental impacts using an attributional LCA approach. By integrating animal performance metrics with feed sustainability indicators, this research seeks to inform balanced strategies for reducing reliance on imported SBM while addressing broader ecological challenges in poultry production systems.

## Materials and methods

All experimental procedures in this study were approved by the Nottingham Trent University School of Animal, Rural and Environmental Sciences ethical review committee, and logged as ARE1790405.

### Animals, diets, and husbandry

A total of 960 one-day male broilers (480 Ross 308 birds and 480 JA787 birds) were randomly distributed into 96 pens with 10 birds/pen. The pens were allocated equally to 2 rearing rooms. The pens were distributed into the following dietary treatments, HN (High nutrient density control +std enzymes), MN (moderate nutrient density control +std enzymes), LEG-STD (UK plant-based protein sources+ standard NSP enzymes), LEG: UK (plant-based protein sources - No NSP enzymes), LEG-S (UK plant-based protein sources + super dose NSP enzymes) (Table 1a, Table 1b). The nutritional composition of the experimental diet is presented in (Table 2). Diets were in crumb format during the starter phase and pellet form for the rest of the trial. The experimental pens were 0.64 m2 in area and bedded on softwood shavings approximately 3 cm in depth. The birds had free access to feed and water during the trial. The standard diets were formulated to meet Ross 308 (Aviagen, 2014) and JA787 (Hubbard, 2023) nutritional requirements.

**Table 1a:** ingredient composition of the starter diets (g/kg).

	HN	MN	LEG	LEG-STD	LEG-S
Wheat	576.4	616.5	467.8	467.8	467.8
Peas Raw Ground	0.0	0.0	75.0	75.0	75.0
Field Beans Ground	0.0	0.0	75.0	75.0	75.0
Lupins 32% Ground	0.0	0.0	75.0	75.0	75.0
Sunflower Extract HI Pro 36%	0.0	0.0	75.0	75.0	75.0
Soy extract HIPRO	342.5	320	160	160	160
L lysine	3.0	2.8	3.5	3.5	3.5
DL methionine	3.8	3.4	3.8	3.8	3.8
L threonine	1.7	1.5	1.6	1.6	1.6
Soya oil	36.0	11.0	30.0	30.0	30.0
Limestone flour	12.5	12.0	12.5	12.5	12.5
Monocalcium Phosphate	11.0	10.0	8.0	8.0	8.0
Salt	2.0	2.0	2.0	2.0	2.0
Sodium Bicarbonate	2.0	1.7	1.7	1.7	1.7
BR. Trial. Sta. PMX. Batch	4.0	4.0	4.0	4.0	4.0
Econase XT 25 Dry	0.05	0.05	0.0	0.05	0.075
Titanium Dioxide	5.0	5.0	5.0	5.0	5.0
Multimite Celite AI Ash	0.0	10	0.0	0.1	0.0
Quantum Blue 5 G Nutrient	0.1	0.1	0.1	0.1	0.5
Signis (Xylanase+Xylooligosaccharide	0.1	0.1	0.0	0.1	0.15

Premix per kg of the diet: Vitamins; A: 8000IU; D3: 5000IU; E:0.075 g; Trace elements; Calcium iodate:3.1 mg (iodine:2.0 mg); Copper sulphate: 80 mg (copper 3b405:27 mg); Iron sulphate: 83.5 mg (Iron 3b103); Zootechnical additives; Axtra PHY 10 000 L: 0.01%; Ronozyme cereal WX-MG MP E1607 in cat N of directive 70/524/EEC: 0.029%; Maxiban G160 (80 mg/kg Narasin and 80 mg/kg Nicarbazin to provide 50 mg/kg Narasin and 50 mg/kg Nicarbazin in final feed as an aid in the prevention of coccidiosis in broiler chickens).

**Table 1b:** ingredient composition of the finisher diets (g/kg).

	HN	MN	LEG	LEG-STD	LEG-S
Wheat	663.7	727.4	576.8	576.6	576.8
Peas Raw Ground	0.0	0.0	60.0	60	60
Field Beans Ground	0.0	0.0	60.0	60	60
Lupins 32% Ground	0.0	0.0	60.0	60	60
Sunflower Extract HI Pro 36%	0.0	0.0	60.0	60	60
Soy extract HIPRO	250	220	110	110	110
L lysine	2.4	2.6	2.6	2.6	2.6
DL methionine	2.9	2.6	2.8	2.8	2.8
L threonine	1.4	1.3	1.2	1.2	1.2
Soya oil	50	18	40	40	40
Limestone flour	9.0	8.0	8.5	8.5	8.5
Monocalcium Phosphate	7.5	7.0	5.0	5.0	5.0
Salt	2.0	2.0	2.0	2.0	2.0
Sodium Bicarbonate	2.0	2.0	2.0	2.0	2.0
BR. Trial. Sta. PMX. Batch	4.0	4.0	4.0	4.0	4.0
Econase XT 25 Dry	0.05	0.05	0.0	0.05	0.075
Titanium Dioxide	5.0	5.0	5.0	5.0	5.0
Quantum Blue 5 G Nutrient	0.1	0.1	0.1	0.1	0.5
Signis (Xylanase+Xylooligosaccharide)	0.1	0.1	0.0	0.1	0.15

Premix per kg of the diet: Vitamins; A: 8000IU; D3: 5000IU; E:0.075 g; Trace elements; Calcium iodate:3.1 mg (iodine:2.0 mg); Copper sulphate: 80 mg (copper 3b405:27 mg); Iron sulphate: 83.5 mg (Iron 3b103); Zootechnical additives; Axtra PHY 10 000 L: 0.01%; Ronozyme cereal WX-MG MP E1607 in cat N of directive 70/524/EEC: 0.029%; Maxiban G160 (80 mg/kg Narasin and 80 mg/kg Nicarbazin to provide 50 mg/kg Narasin and 50 mg/kg Nicarbazin in final feed as an aid in the prevention of coccidiosis in broiler chickens).

**Table 2:** Nutritional composition of the experimental diets.

		Starter			Finisher	
	HN	MN	LEG	HN	MN	LEG
Oil EE (%)	5.15	2.69	4.81	6.57	3.44	5.78
Protein (%)	22.6	21.9	21.6	19	18.3	18.5
Fibre (%)	2.87	2.91	5.21	2.8	2.9	4.69
ME (MJ/kg)	12.7	12.1	12.1	13.4	12.8	12.8
T-Lysine (%)	1.47	1.4	1.4	1.17	1.12	1.11
D-lys (%)	1.29	1.22	1.22	1.02	0.969	0.964
M+C (%)	1.05	0.991	1	0.862	0.813	0.823
DM+C (%)	0.97	0.92	0.917	0.808	0.767	0.763
Threo (%)	0.994	0.943	0.934	0.814	0.766	0.765
D-Thr (%)	0.841	0.797	0.793	0.692	0.653	0.651
Trypt (%)	0.272	0.263	0.241	0.226	0.214	0.204
D-Try (%)	0.232	0.225	0.198	0.194	0.185	0.17
Val (%)	1.02	0.988	0.875	0.855	0.819	0.753
ILe (%)	0.856	0.8	0.678	0.625	0.55	0.498
Arg (%)	1.53	1.47	1.43	1.25	1.17	1.18
Ca (%)	1.021	0.979	0.975	0.798	0.743	0.742
P (%)	0.655	0.627	0.609	0.533	0.517	0.498
Av P (%)	0.499	0.475	0.492	0.406	0.394	0.399
Na (%)	0.192	0.184	0.178	0.191	0.193	0.188
K (%)	0.944	0.917	0.9	0.799	0.767	0.775

The lightning system in the rearing rooms started by 1 h of darkness then increased each day by 1 hour up to 6 h maintained from d 6 to 21. The temperature was set at 32°C on D1 which was then decreased by 0.5°C every until it reached 21.5°C then maintained for the rest of the trial.

Bird weight and feed intake (feed offered – feed remaining) were recorded for each pen at the end of each developmental stage, starter (D10 for Ross 308 and D14 for JA787), grower (D24 for Ross 308 and D27 for JA787), and finisher (D35 for Ross 308 and D45 for JA787). One bird per pen was randomly sampled (D35 for Ross 308 and D43 for JA787) and total meat yield was recorded as the sum of breast, thigh, and drumstick meat.

### Life cycle analysis

Life cycle inventory (LCI) data for the experimental feed were obtained from the Global Feed LCA Database using the ReCiPe 2016 midpoint (H) method with mass allocation (de Boer, 2021) (Table 3). Pesticide freshwater ecotoxicity was estimated at a screening level using a generic USEtox-based characterisation factor of 2,000 kg 1,4-DCB eq/ kg and a default emission fraction to freshwater of 2% of the applied active ingredient (Rosenbaum et al., 2008). This approach was applied for comparative screening purposes only and does not constitute a pesticide-specific risk assessment. Environmental impacts associated with feed consumption were calculated at the pen level, with one kg of meat defined as the functional unit. Environmental impacts not related to feed consumption were excluded from the analysis. The system boundary for all LCA data was cradle-to-farm gate. Two LCI datasets were developed: a baseline scenario and a scenario assuming a 30% reduction in synthetic fertiliser and plant protection inputs, implemented by modifying input parameters in GFLI Feedprint International app.

**Table 3:** Effect of legume and enzymatic supplementation on the overall growth performance of fast and slower growing broiler.

JA787	D0 bird weight (g)	D42 bird weight (g)	Weight gain (g)	Feed intake (g)	Meat yield (g)
HN	35.8	2696a	2660a	4312	1093
MN	35.3	2689ab	2653ab	4303	1075
LEG	35.3	2564b	2528b	4393	1034
LEG-STD	35.5	2614ab	2578ab	4433	1050
LEG-S	35.7	2640ab	2604ab	4405	1124
					
Ross308					
HN	40.2	2897	2856a	4013	1221
MN	40.3	2908	2868a	4129	1201
LEG	40	2793	2754a	4070	1225
LEG-STD	40.4	2868	2829a	4097	1230
LEG-S	40.5	2887	2846a	4070	1323
Main effect					
HN	38			6318.5	1703.5
MN	37.8			6367.5	1675.5
LEG	37.65			6428	1646.5
LEG-STD	37.95			6481.5	1665
LEG-S	38.1			6440	1785.5
					
SEM	0.342	46.2	0.072	58.4	47
Diet	0.784	0.072	<0.001	0.498	0.409
Strain	<0.001	<0.001	<0.001	<0.001	<0.001
Diet*strain	0.88	<0.001	0.072	0.605	0.89

HN: High nutrient density control +std enzymes, MN: Moderate nutrient density control +std enzymes, LEG: UK plant-based protein sources - No NSP enzymes, LEG-STD: UK plant-based protein sources+ standard NSP enzymes, LEG-S: UK plant-based protein sources + super dose NSP enzymes. The standard enzyme mix was 0.01% phytase and 0.01% Signis (Xylooligosaccharide and xylanase) and super dose enzyme mix was 0.05% phytase and 0.015% Signis.

### Statistical analysis

The trial data were analysed using analysis of variance according to the following model:

$$Y_{\text{ijkl}}=M+\text{TR}T_{i}+\text{Strai}n_{j}+\text{TRT}*\text{Strai}n_{\text{ij}}+\text{Roo}m_{k}+\text{Bloc}k_{l}+E_{\text{ijkl}}$$

Where Y_ijkl_, M, TRT_i_, Strain_j_, TRT*Strain_i_, Room_k_, Block_l_, and E_ijkl_ were response variables, overall mean, effect of diet, effect of broiler strain, effect of the interaction between the treatment and bird strain, effect of room, effect of block, and the residual, respectively. Means were compared using Fisher LSD with Bonferroni correction. R was used to perform data analysis (R core Team, 2024).

## Results

### Growth performance and meat yield

Bird strain had significant effect on final bird weight, feed intake, or meat yield (P < 0.001) (Table 4). There was significant effect of the treatment, bird strain, and treatment-bird strain interaction on bird weight gain (Table 3). There was significant effect of diet*bird strain interaction on final bird weight (P < 0.001). The experimental diets did not significantly differ in feed intake or meat yield for both strains of birds (P > 0.05). legume based diets with enzymatic supplementation (regardless of the supplementation level) did not significantly differ from HN and MN diets regardless of the bird strain (P > 0.05).

**Table 4:** Life cycle analysis inventory of the experimental feeds.

	Source	GWP	EUT	ACID	LU	WRD	ETOX
*Conservation produced*							
Peas raw ground	UK	330	32.6	7.36	32.9	4.9	9.31
Field beans ground	UK	316	27.7	6.32	27.3	4.4	15.9
Lupins 32% ground	UK	1458	91.3	21.2	37.2	16.9	141
							
*Conventionally produced*							
Wheat raw enz (ME+3%)	UK	327	41.7	9.4	9.1	11	13.4
Peas raw ground	UK	352	32.9	7.43	39.2	5.6	13.3
Field beans ground	UK	361	29.6	6.76	27.3	5.6	22.7
Lupins 32% ground	GER	1518	91.9	21.3	73.2	18.6	201
Sunflower ext.hi pro 36%	AR	733	33.5	8.67	60.6	1260	39.7
Soya Ext Hipro	BR	6250	42.8	10.6	39.8	104	27
L-Lysine batch	NETH	6487	145	53.7	19.1	2040	0
DL-methionine batch	NETH	3044	27.1	13.8	0	405	0
L-Threonine batch	NETH	6487	145	53.7	19.1	2040	0
Soya oil	NETH	6940	47.9	12.2	45.5	12000	27
Limestone flour tru.270	NETH	1219	6.81	2.5	0	15100	0
Monocalcium phosphate	NETH	569	9.68	3.18	0	11.5	0
Salt	NETH	174	1.69	1.3	0	42.9	0
Sodium bicarbonate	NETH	485	15.2	5.49	0.4	414	0
Br trial STA BMX batch	NETH	6449	139	51.8	17.9	1980	0
Econase XT 25 dry	NETH	2000	286	109	38.2	4630	0
Titanium dioxide	NETH	0	0	0	0	0	0
Multimite celite AI ash	NETH	0	0	0	0	0	0
Quantum blue 5 g nutrient	NETH	2000	286	109	38.2	4630	0

GWP: Global warming - including land use change (kg CO2 eq/t), ACID: Terrestrial acidification (mol H+ eq /t), EUT: Freshwater eutrophication (Mol N eq/t), ETOX: Freshwater ecotoxicity (kg 1,4 DCB/t), LU: Land use (ha·a crop eq /100 t), WRD: Water resource depletion (l/100 t).

### Feed ingredient LCA inventory

Lupin exhibited substantially higher environmental impacts than pea and bean, with values approximately three to four times greater GWP, EUT, ACID, LU, and WRD (Table 4). Notably, lupin showed an order‑of‑magnitude increase (≈10‑fold) in ecotoxicity (ETOX) compared with the other legumes. A 30% reduction in synthetic fertiliser application and plant protection inputs resulted in marked decreases in environmental impacts across all crops, although the magnitude of response varied by crop and impact category.

In pea, the same management change reduced GWP by 6.25%, LU by 16%, WRD by 12.5%, and ETOX by 30%. For bean, reductions were 12.5% for GWP, 6.5% for both EUT and ACID, 21% for WRD, and 30% for ETOX. In contrast, lupin showed a relatively small reduction in GWP (4%) but a pronounced decrease in LU (49%), alongside reductions of 9% in WRD and 30% in ETOX.

### Life cycle analysis for commercial and legume-based diets

Fig. 1, Fig. 2 shows the relative contribution of feed ingredient to environmental impact (per one functional unit) of HN, MN, and LEG diets.

**Fig. 1:**
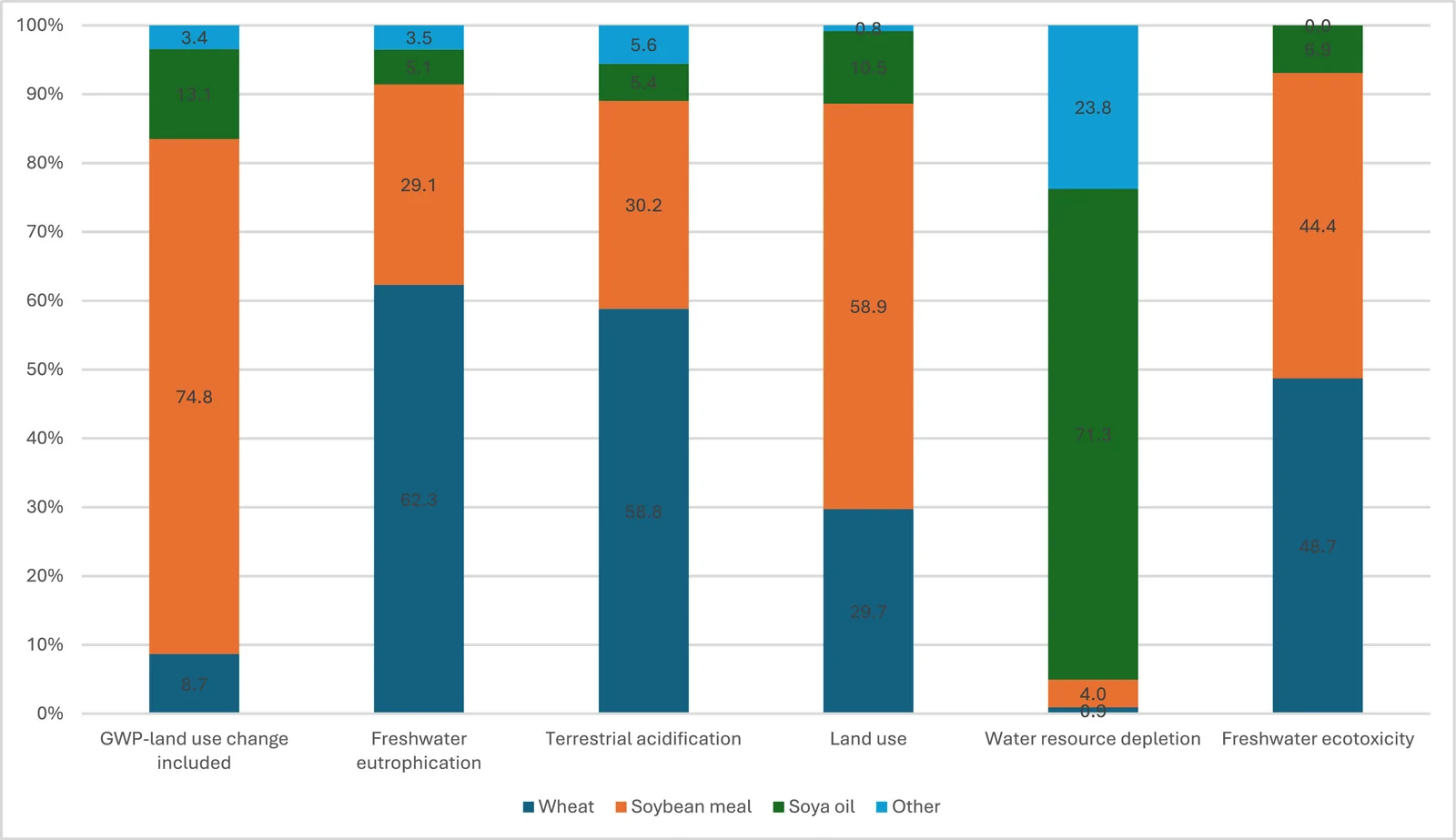
Relative contribution (%) of feed ingredient to the environmental impact of standard broiler diet consumed by broilers.

**Fig. 2:**
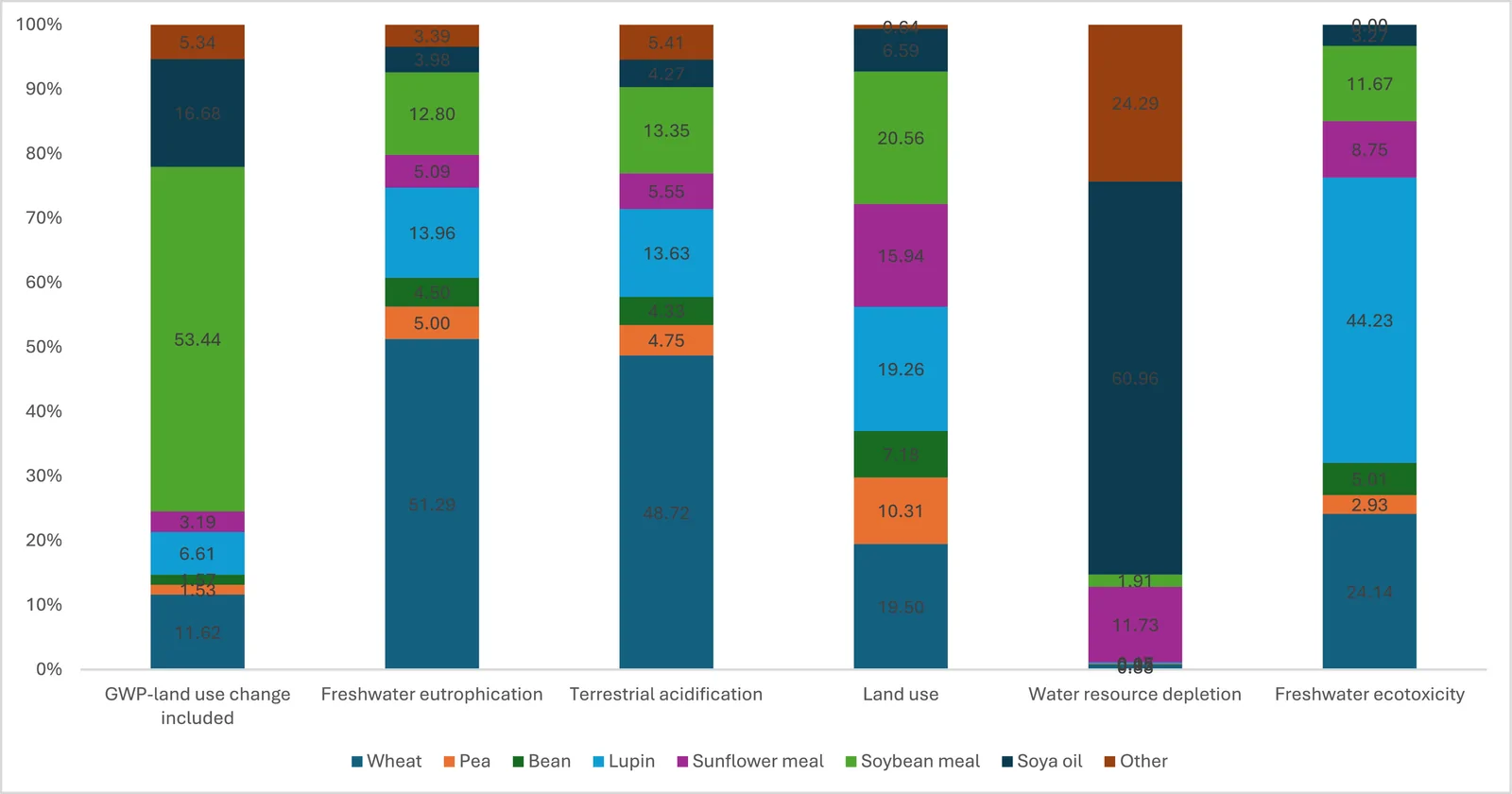
Relative contribution of feed ingredient to the environmental impact of legume-based diet consumed by broilers.

In HN and MN diets, soybean meal was the dominant contributor to GWP, accounting for approximately 75% of the total impact followed by soya oil (14%) and wheat (5%). Freshwater eutrophication and ACID were driven primarily by wheat, which accounted for 62% of the total impact, with soybean meal contributing a further 29%. Land use was mainly associated with soybean meal (59%) and wheat (30%). Water resource depletion was largely attributed to soya oil (71%) and feed additives (24%). Freshwater ecotoxicity was driven predominantly by wheat (48%) and soybean meal (44%).

In the legume‑based diet, SBM and soya oil contributed to 53% and 17% of total GWP, respectively. Wheat and SBM together accounted for a substantial share of GWP, contributing 30% and 22%, respectively. Wheat was the principal contributor to EUT and ACID (51%), followed by lupin (13%) and SBM. Land use was evenly distributed, with SBM, wheat, and lupin each contributing approximately 20% of the total. Water resource depletion was dominated by soya oil (61%), followed by additives (24%) and sunflower meal (12%). Freshwater ecotoxicity was mainly associated with lupin (44%), wheat (24%), and soybean meal (12%).

### Life cycle analysis: conventional legume production scenario

There was significant effect of the diet on GWP, ACID, EUT, ETOX, LU, and WRD (P ≤ 0.05) (Table 5). The effect of bird strain on GWP, LU, WRD, and ETOX was significant (P ≤ 0.05). The treatment*strain interaction significantly affected GWP-LC, EUT, ACID, and LU.

**Table 5:** The effect of conventionally grown legume and enzymatic supplementation on environmental impact of broilers.

JA787	GWP	EUT	ACID	LU	WRD	ETOX
HN	8.96(a)	0.192(ab)	0.046(ab)	8.37(b)	3.32	69.2
MN	8.06(b)	0.178(b)	0.042(b)	7.3(c)	1.66	67.4
LEG	6.01(c)	0.207(a)	0.049(a)	11.3(a)	3.34	127
LEG-STD	5.94(c)	0.205(a)	0.048(a)	11.2(a)	3.31	128
LEG-S	5.55(c)	0.191(ab)	0.045(ab)	10.5(a)	3.09	118
						
Ross308						
HN	7.41(a)	0.16(a)	0.038(a)	6.97(b)	2.79	57.7
MN	6.82(a)	0.153(a)	0.036(a)	6.23(b)	1.44	57.7
LEG	4.66(b)	0.162(a)	0.038(a)	8.83(a)	2.63	100
LEG-STD	4.66(b)	0.162(a)	0.038(a)	8.83(a)	2.63	99.9
LEG-S	4.42(b)	0.154(a)	0.036(a)	8.37(a)	2.49	94.7
						
Main effect						
HN					3.06(a)	98.1(b)
MN					1.55(b)	96.3(b)
LEG					2.99(a)	177(a)
LEG-STD					2.97(a)	178(a)
LEG-S					2.79(a)	165(a)
SEM	0.301	0.008	0.002	0.388	0.12	4.06
P						
Treatment	<0.001	<0.001	<0.001	<0.001	<0.001	<0.001
Strain	<0.001	0.112	0.116	<0.001	<0.001	<0.001
Treatment*strain	0.009	0.02	0.05	0.025	0.267	0.055

GWP: Global warming- including land use change (kg CO2 eq/kg meat), ACID: Terrestrial acidification (mol H+ eq /kg meat), EUT: Freshwater eutrophication (Mol N eq/kg), ETOX: Freshwater ecotoxicity (kg 1,4 DCB/t meat), LU: Land use (ha·a crop eq /10 t meat), WRD: Water resource depletion (l/100 kg meat). HN: High nutrient density control +std enzymes, MN: Moderate nutrient density control +std enzymes, LEG: UK legume - No NSP enzymes, LEG-STD UK legumes + standard NSP enzymes, LEG-S: UK plant-based protein sources + super dose NSP enzymes. The standard enzyme mix was 0.01% phytase and 0.01% Signis (Xylooligosaccharide and xylanase) and super dose enzyme mix was 0.05% phytase and 0.015% Signis.

Both LEG and LEG-STD had significantly higher EUT and ACID compared to MN for JA787 birds (P ≤ 0.05) while diets did not significantly differ in Ross 308 birds (P > 0.05). JA787 birds fed MN diet had significantly lower LU compared to HN (P ≤ 0.05). Inclusion of legume in the diet regardless of the enzymatic treatment significantly increased LU (P ≤ 0.05) for both bird strains. Both JA787 and Ross 308 birds fed MN had significantly lower WRD compared to HN (P ≤ 0.05) while the inclusion of legume did not significantly change WRD. Replacing SBM with legume significantly increased ETOX compared to HN and MN regardless of the bird strain or enzymatic supplementation (P ≤ 0.05) (Table 6).

**Table 6:** The effect of conservation-grown legume and enzymatic supplementation on environmental impacts of broilers.

JA787	GWP	EUT	ACID	LU	WRD	ETOX
HN	8.96(a)	0.192(ab)	0.046(ab)	8.37(b)	3.32	69.3
MN	8.06(b)	0.178(b)	0.042(b)	7.3(c)	1.67	67.4
LEG	5.91(c)	0.204(a)	0.048(a)	10(a)	3.31	107
LEG-STD	5.97(c)	0.206(a)	0.049(a)	10.1(a)	3.34	108
LEG-S	5.51(c)	0.19(ab)	0.045(ab)	9.36(ab)	3.09	99.8
						
Ross308						
HN	7.41(a)	0.16(a)	0.038(a)	6.97(ab)	2.79	57.7
MN	6.82(a)	0.153(a)	0.036(a)	6.23(b)	1.44	57.7
LEG	4.64(b)	0.162(a)	0.038(a)	7.91(a)	2.63	84.3
LEG-STD	4.63(b)	0.162(a)	0.038(a)	7.9(a)	2.62	84.3
LEG-S	4.39(b)	0.153(a)	0.036(a)	7.49(a)	2.49	79.9
						
Main effect						
HN					3.06(a)	63.5(b)
MN					1.56(b)	62.6(b)
LEG					2.97(a)	95.7(a)
LEG-STD					2.98(a)	96.2(a)
LEG-S					2.79(a)	89.9(a)
SEM	0.301	0.008	0.002	0.363	0.12	3.6
P						
Treatment	<0.001	<0.001	<0.001	<0.001	<0.001	<0.001
Strain	<0.001	0.134	0.136	<0.001	<0.001	<0.001
Treatment*strain	0.008	0.008	0.006	0.043	0.267	0.159

GWP: Global warming- including land use change (kg CO2 eq/kg meat), ACID: Terrestrial acidification (mol H+ eq /kg meat), EUT: Freshwater eutrophication (Mol N eq/kg), ETOX: Freshwater ecotoxicity (kg 1,4 DCB/t meat), LU: Land use (ha·a crop eq /10 t meat), WRD: Water resource depletion (l/100 kg meat). HN: High nutrient density control +std enzymes, MN: Moderate nutrient density control +std enzymes, LEG: UK legume - No NSP enzymes, LEG-STD UK legumes + standard NSP enzymes, LEG-S: UK plant-based protein sources + super dose NSP enzymes. The standard enzyme mix was 0.01% phytase and 0.01% Signis (Xylooligosaccharide and xylanase) and super dose enzyme mix was 0.05% phytase and 0.015% Signis.

### Life cycle analysis: conservative/low input legume production scenario

There was significant effect of the diet on GWP, EUT, ACID, LU, WRD, and ETOX (P ≤ 0.05) (Table 6). Bird strain had significant effect on GWP, LU, WRD, and ETOX (P ≤ 0.05). The effect of bird strain*diet interaction on GWP, EUT, ACID, and LU was significant (P ≤ 0.05). Replacing SBM with legume significantly reduced GWP regardless of the bird strain (P ≤ 0.05). Both LEG and LEG-STD significantly increased EUT and ACID of JA787 birds (P ≤ 0.05) while the diet did not significantly alter EUT or ACID of Ross 308 birds (P > 0.05). Soybean meal replacement with legume significantly increased LU for both strains for birds (P ≤ 0.05) regardless of the enzymatic treatment. Replacing SBM with legume did not reduced WRD compared to HN (P ≤ 0.05). The ETOX in legume diets was significantly higher than that of SBM diets regardless of the bird strain or enzymatic treatment (P ≤ 0.05).

## Discussion

Replacing imported soybean meal (SBM) with locally produced legumes has been increasingly advocated as a key mitigation strategy to support the UK’s transition towards net-zero agriculture, due to its potential to reduce both embedded and domestic greenhouse gas (GHG) emissions (Bowels, 2026). The UK livestock sector is highly dependent on imported soy, predominantly from South America, where production is closely associated with deforestation, land-use change, and substantial carbon emissions (Bowels, 2026). Substitution with home-grown protein sources such as peas, beans, or lupins can substantially reduce these emissions by shortening supply chains and avoiding deforestation-linked carbon costs, while also delivering system-level benefits through biological nitrogen fixation and reduced reliance on synthetic fertilisers (Bowels, 2026).

While greenhouse gas emissions and global warming potential (GWP) remain the most frequently reported environmental indicators in livestock systems, they represent only one dimension of sustainability. Life cycle assessment (LCA) considers a broader range of environmental impacts, including eutrophication potential (EUT), which reflects nutrient pollution of aquatic ecosystems; acidification potential (ACID), associated with emissions that contribute to soil and water acidification; land use (LU); water resource depletion (WRD); and ecotoxicity (ETOX), which reflects the potential for chemical emissions to adversely affect terrestrial and aquatic environments. Consequently, dietary interventions that reduce GWP do not necessarily improve performance across all environmental indicators, highlighting the importance of evaluating multiple impact categories when assessing feed sustainability.

Partial replacement of SBM with local protein sources has been shown in previous studies to sustain growth performance while reducing GWP of broilers (Fatica et al., 2022); (Matton et al., 2025). However, the effect of these substitution strategies on other environmental impact categories have rarely been evaluated. The present results demonstrate that changes in diet composition do not necessarily result in consistent responses across all environmental indicators, with some feed ingredients exhibiting favourable outcomes for certain impact categories but not others. Nevertheless, the production of legumes under reduced fertilisation and plant protection regimes appeared to improve environmental performance across multiple impact categories simultaneously, suggesting that crop management practises may be as important as ingredient selection when seeking to improve the overall sustainability of broiler diets.

Therefore, the goal of the current study was to determine the effect of partial SBM substitution with locally produced legumes on broiler environmental impact using multi-dimensional LCA and if enzymatic supplementation and conservation cropping could reduce the trade-offs between the environmental impact categories.

The results of the current study showed that partial replacement of SBM with UK‑grown legumes and sunflower meal can deliver meaningful reductions in feed‑related GWP without compromising broiler performance or carcass yield regardless of the bird strain.

This is a direct result of displacing SBM (which has high weight on GWP) with home grown legumes while maintaining feed intake, growth, and meat yield of birds. Furthermore, this benefit became clearer under the conservative/low‑input crop production scenario. This outcome aligns with the broader feeding literature showing that legumes can replace a portion of SBM without major losses when diets are carefully balanced for digestible amino acids and energy (Biesek et al., 2020). Therefore, UK legume integration in replacement of SBM can be a credible decarbonisation lever for poultry feed but only as one element of a broader strategy because carbon benefits alone do not guarantee overall environmental improvement. Indeed, the current study demonstrates these climate gains were accompanied by trade‑offs most notably higher LU and elevated, WRD, and ETOX.

The determinants of these undesirable changes in certain categories of environmental impact are multifactorial. In LEG diets, SBM was partially replaced with lupin and sunflower meal which has 1.8 times and 1.5 times LU compared to SBM, which could explain the increase in LU of broilers fed LEG diets. However, both EUT and ACID were not increased when SBM was partially replaced by legumes and sunflower meal. Two factors contribute to this outcome: firstly, the EUT and ACID impact of SBM is already comparable to legumes and sunflower meal. The second and most influencing factor is that wheat rather than dietary plant protein sources was the main contributor to EUT and ACID the standard broiler diet.

The most persistent trade‑off in this study was ETOX, which increased significantly in legume diets across strains and was not reduced under the conservative/low‑input scenario. Ingredient contribution results and crop inventories in the dataset indicate that lupin carried substantially higher ETOX potential than pea and bean, and it became a major contributor to diet‑level ETOX, explaining why overall ecotoxicity remained elevated even when some agronomic inputs were reduced.

The NSP enzyme supplementation in the current study did not materially change LCA results across impact categories. Mechanistically, xylanase and related carbohydrases can reduce digesta viscosity, release encapsulated nutrients, and modulate hindgut fermentation through production of oligosaccharides, with demonstrated effects on growth and fermentation metabolites in some broiler studies. However, in a feed‑consumption LCA normalised to meat yield, enzymes can only lower impacts if they measurably improve performance (e.g., reduce feed intake per kg meat) or enable formulation changes that displace high-impact ingredients, and those pathways were not strongly expressed here because feed intake and carcass yield were largely unchanged across enzyme treatments. The absence of detectable LCA benefit from enzymes is therefore consistent with both LCA logic and the broader enzyme literature showing that responses are context‑dependent and may not translate into improved feed efficiency under all diet compositions and baseline digestibility conditions (Kocher et al., 2000); (Singh et al., 2021).

The findings reinforce a key sustainability insight echoed in global food-system syntheses. Interventions can reduce climate impacts while increasing other pressures, and decision‑making should therefore be guided by multi‑indicator evidence rather than single‑metric optimisation. The study also illustrates that feed crop management scenarios matter. The conservative/low‑input legume production reduced several crop‑level impacts and strengthened the climate benefit of legume substitution, implying that coordinated changes in agronomy and feed formulation are more likely to deliver balanced outcomes than ingredient substitution alone.

Ingredient inventories may embed regional assumptions (e.g., non‑UK production data for some ingredients) that influence category results, particularly for land use and pesticide‑related categories, which again points to the value of UK‑specific primary inventories. Thus, it is important to develop high‑resolution and open access UK life cycle inventories for peas, faba beans, and lupins using primary farm data and uncertainty analysis, because representativeness can strongly influence trade‑off conclusions.

Given the strong contribution of individual ingredients to specific categories (e.g., lupin to ecotoxicity in this dataset), formulation work should test whether rebalancing the legume mix or replacing high‑ETOX fractions can retain climate benefits while reducing the overall toxicity burden.

Overall, UK legume substitution offers a realistic, performance‑compatible route to reduce feed‑related GWP, but it does not automatically deliver “across‑the‑board” sustainability improvements, because LU and ETOX trade‑offs can persist and may require targeted agronomic innovation, better inventories, and multi‑criteria optimisation across crop production and formulation.

Growth performance and meat yield were included to establish whether the environmental effects were associated with changes in productive output and to support the calculation of environmental impacts per unit of meat produced. These variables are therefore interpreted in the context of the environmental assessment rather than as a comprehensive investigation of broiler growth responses.

## Conclusion

The findings of the current study underscore the complexity of sustainability decisions in poultry nutrition. Partial substitution of imported soybean meal with UK-grown legumes, can deliver meaningful reductions in feed-related GWP without compromising broiler growth performance or carcass yield. However, these gains in climate impact mitigation were accompanied by trade-offs in other environmental dimensions, notably increased LU, WRD, and ETOX. However, conservative production of faba bean, pea, and lupin is promising in decreasing that tension and brining more holistic environmental benefits.

Ultimately, achieving genuinely sustainable feed systems will require coordinated interventions spanning crop production and feed formulation rather than single-factor substitutions. Further research should identify the optimal balance among pea, faba bean, lupin and sunflower meal using multi-objective formulation that integrates nutritional adequacy, animal performance, cost and environmental impacts.

The findings of this study have implications that extend beyond poultry nutrition, offering insights for any sector employing attributional LCA to guide sustainability decisions.

## Author contributions

**Ashraf Alkhtib** was responsible for conceiving and designing the study, developing the methodology, conducting the investigation, curating and analysing the data, interpreting the findings, preparing the figures and tables, and drafting and revising the manuscript. **Dawn Scholey** contributed to conceiving and designing the study, supervising the research, interpreting the findings, and critically reviewing and revising the manuscript for important intellectual content. **Mike Bedford** and **James Bentley** contributed to interpreting the findings, refining the scientific content, and critically reviewing and revising the manuscript. James Bentley contributed to interpreting the findings and critically reviewing and revising the manuscript for important intellectual content. **Emily Burton** contributed to conceiving and designing the study, securing funding, supervising the research, interpreting the findings, and critically reviewing and revising the manuscript.

All authors made substantial intellectual contributions to the conception, design, analysis or interpretation of the work. They were involved in drafting the manuscript or critically revising it for important intellectual content, reviewed and approved the final version submitted for publication, and agree to be accountable for all aspects of the work. They will ensure that any questions concerning the accuracy or integrity of any part of the article are appropriately investigated and resolved.

## Disclosures

The authors declare that they have no known financial or personal conflict of interests.

## Data Availability

Data of the study will be available by the corresponding author on a reasonable request.
